# Supported employment: the fundamental adjuvant in the treatment of mental illness

**DOI:** 10.1192/j.eurpsy.2023.245

**Published:** 2023-07-19

**Authors:** J. R. P. Correia, H. J. Gomes, R. A. Moreira, A. M. Fraga, S. Neves, J. R. Gomes

**Affiliations:** 1 Departamento de Psiquiatria e Saúde Mental, Unidade Local de Saúde do Nordeste, Bragança; 2 Departamento de Psiquiatria e Saúde Mental, Unidade Local de Saúde do Nordeste, Bragança, Portugal; 3 Departamento de Psiquiatria e Saúde Mental, Hospital de Cascais, Cascais; 4 Centro Hospitalar de Tâmega e Sousa, Penafiel, Portugal

## Abstract

**Introduction:**

Recent scientific evidence confirms that employability is extremely important in mental health care. Employment promotes a healthy lifestyle and unemployment leads to a global deterioration in health. This principle is transversal to all areas of health, applying equally to people with mental illness, including serious mental illness such as schizophrenia and bipolar affective disorder.

**Objectives:**

Highlight the importance of employability in the treatment and rehabilitation process of people with mental illness.

**Methods:**

PubMed database searched using the terms “supported employment” and “mental health” and “policies”.

**Results:**

Parallel to conventional psychiatric treatments, employment generates self-confidence, promotes social responsibility, a sense of belonging and, consequently, integration in the community. From an economic point of view, it brings financial autonomy to the sick person, allowing the financing of their own accommodation, the payment of proposed treatments and the enjoyment of structures and leisure activities that until then would be impossible. It is also known that patients who are employed are less likely to resort to psychiatric emergency services and have a lower rate of readmissions to psychiatric hospitals, reflecting a better ability to manage the disease. Overall, employability increases the sick person’s quality of life, not only being an effective short-term treatment, but also one of the only interventions that reduce dependence on the health system in the long term.

**Conclusions:**

The treatment plan should aim for more than the suppression of symptoms.

Knowing that employment generates positive outcomes, gets that as fundamental parameter for the treatment and for the rehabilitation of the person with mental illness, and it must therefore become essential that mental health services help patients to find satisfactory jobs and that protect your needs.

Thus, mental health policies should defend a new mental health treatment paradigm and emphasize employment as an imperative measure in the treatment and psychosocial rehabilitation of the sick person, including supported employment as an essential part of treatment.

**Disclosure of Interest:**

None Declared

